# Factors influencing exclusive breastfeeding duration in Pakistan: a population-based cross-sectional study

**DOI:** 10.1186/s12889-021-12075-y

**Published:** 2021-11-03

**Authors:** Sidra Arif, Hina Khan, Muhammad Aslam, Muhammad Farooq

**Affiliations:** 1 Crop Reporting Service, Agriculture Department Punjab, Jhang, Pakistan; 2 Department of Statistics, GC University Lahore, Lahore, Pakistan; 3 Department of Statistics, Faculty of Science, King Abdulaziz University, Jeddah, 21551 Saudi Arabia

**Keywords:** Exclusive breastfeeding, Pakistan, Binary logistic regression analysis, Survival analysis, Demographic and social factors

## Abstract

**Background:**

Breastfeeding has the most profound impact on infant health and wellness, and also have significant implications for the mother. The duration of the breastfeeding determines the infant’s protection from malnutrition and other common infectious diseases; consequently, the World Health Organization (WHO) recommends exclusive breastfeeding (EBF) six months, followed by gradual weaning and breastfeeding until the baby is two years old. In Pakistan, the practice of breastfeeding is heavily dependent upon certain demographic, economic, social, and biological factors, which ultimately impact the quality of care provided to the infant and their health. The aim of this paper, therefore, is to measure the impact of these factors on the exclusive breastfeeding duration in Pakistan.

**Methods:**

The data for the study has been collected from Pakistan Demographic and Health Survey (PDHS) for the year 2017–18. Binary logistic regression model and survival analysis are used to determine the relationship between the independent and dependent variables.

**Results:**

We use a binary logistic regression to estimate the effect of each factor on the duration of EBF. The binary logistic regression finds significant relationships between region, maternal education, wealth index, size of a child, watching television, delivery by cesarean, and maternal age and EBF. We then use log-likelihood, AIC, BIC criteria to determine if a parametric or non-parametric model would provide a better fit; based on these results we fit an Inverse Gaussian (Weibull) distribution for the survival analysis. These results show that there are more significant factors associated with EBF duration in parametric survival analysis than in the binary logistic regression results. Thus, the survival analysis is a better method for predicting the relationship between the duration of EBF and its factors. Furthermore, logically EBF is designated to be done for six months which would not be properly gauged with a binary response variable.

**Conclusions:**

The results of this study provide proof that exclusive breastfeeding is a common practice among women in Pakistan, and to improve the quality of post-natal care, health policy in the country needs to focus on the existing demographic and social factors which are found significant in this study.

**Supplementary Information:**

The online version contains supplementary material available at 10.1186/s12889-021-12075-y.

## Background

Within the South Asian region, the conditions regarding infant health are particularly dire, and some of the most common inflictions on infant and child health in this region include malnutrition, stunting, and pneumonia. Such problems are exacerbated using artificial feeding for infants and lead towards long-term complications such as obesity, diabetes, and higher blood pressure [1]. In this regard, breastfeeding is championed as an essential and integral part of infant health and is a proven measure for improved immunity, growth and early development among children. Additionally, exclusive breastfeeding (EBF) provides improves survival odds for infants, where children who are breastfed are fourteen times more likely to survive when compared to children who have not been breastfed, and prevents diarrhea and pneumonia, which are the two leading causes of infant death [2].

However, despite this favorable evidence, there is a marked reluctance and underuse of exclusive breastfeeding practices in Pakistan. There are several reasons for this; the most significant of which are cultural, economic, and socio-economic factors that discourage mothers from continuing the practice of exclusive breastfeeding. Statistically, in Pakistan, only 18% of women start the process of initial breastfeeding and only 37.7% of the women continue the practice of exclusive breastfeeding [3]. Such statistics pose dismal odds for infant health and highlight an issue that needs to be investigated so that the contributors for a lack of EBF practices can be known. In contrast, EBF decreases infant mortality, increases intelligence and brain growth, decreases coughing, decreases the risk of diabetes helps to fight respiratory disease, enhances mental and physical capability, fights against gastrointestinal infections, enhances learning powers, and lessens obesity risks [4]. In Pakistan, there is dire need to consider the maternal socioeconomic status and peer counseling in order to enhance EBF. Government should impose restrictions on the marketing of formula milk for infants and promote breastfeeding practices through mass media campaign [5]. The literature for EBF practice is full of the relationship between infant health and EBF practices yet there is a dearth of studies on identifying the exact list of factors that contribute the most to a lack of EBF practices in Pakistan.

The issues of improper feeding practices within Pakistan and its neighboring developing countries have been addressed many times in the academic literature and the topic has also been highlighted on several important forums. Most of the studies on this topic have identified the relationship between the EBF duration and its impacts on child health and nutrition and use a variety of statistical methods for doing so. For instance, a study investigating the impact of media campaigns on breastfeeding behaviors within Sindh, Pakistan was conducted by Kim et al. [5],; this case study investigated breastfeeding practices in the province of Sindh after a media campaign was launched to promote healthy breastfeeding practices. The results of the study confirmed that the media campaign was ineffective to combat the existing unhealthy breastfeeding practices in Sindh; this was mainly because of the set cultural, social, and political factors which strongly influence this behavior. Similar cases for unhealthy infant feeding practices can be found in similar undeveloped countries and developing countries in the South-Asian region as well as in Africa. In India, early termination of breastfeeding, suboptimal level of breastfeeding initiation and exclusive breastfeeding were found to base on urbanicity, higher level of mother education and higher household socioeconomic position [6]. Improvement in EBF prevalence increased per capita total cumulative non-health GDP significantly and decreased under 5-mortality rate, reduction of economic cost found in a meta-analysis of sub-saharan Africa [7]. Nigerian study identified some of the major factors which are the culprits for non-EBF practices and highlighted that the significant factors associated with non-EBF practices were a low rate of maternal education, lesser availability of antenatal care, poor to middle household wealth, and lesser availability and awareness for maternal care after birth. The results of this study also suggested the need to study such factors in differing context-specific settings [8].

Therefore, it is important to use statistical means to estimate the impact of each contributing factor on the practice of EBF. Some of these factors include limited financial resources, maternal education and information, region, age, and gender roles and responsibilities. We use a total of nineteen factors and determine the impact of each using binary regression model as well as survival parametric estimations of EBF duration and the selected factors. Thus, the purpose of this paper is to identify significant factors on EBF practice within Pakistan, using data provided by the Pakistan Demographic Health Survey 2017–2018.

Several studies on factors influencing breastfeeding are available in the literature. By exploring the literature and according to the best of authors’ acknowledge, there is no study on factors affecting breastfeed that are considered in this paper. Most recent and reliable data of national- level survey based on international standards is used with a focus on exclusive breastfeeding which is on priority on WHO agenda to prevent under 5-mortality and disease. Exclusive breastfeeding is a time span till no weaning started however WHO recommended standard is for six months; we have applied survival analysis also to undertake the nature of variable to gauge significant impact factors truly, which is never been used before. Findings will add to meet SDG target 3 to promote collective well-being and public health. The limitation of the study is to consider 4 major regions of Pakistan: Punjab, Sindh, Baluchistan, and KPK, out of 8 regions included in PDHS 2017–18.

## Methods

### Study design

We used a cross-sectional study design and user data which was collected by the Pakistan Demographic Health Survey (PDHS) 2017–2018. The Pakistan Demographic Health Surveys are nationwide household surveys that provide a wide range of indicators in the fields of health, nutrition, and population [9]. The surveys are completed with help from the United States Agency for International Development (USAID), alongside technical assistance from the Pakistan Bureau of Statistics (PBS) and ICF International. To date, there have been four PDHS Surveys, and have been completed in the years 1990–1991, 2006–2007, 2012–2013, and lastly, 2017–2018.

### Data collection

We use data from the PDH 2017–2018 survey; this survey is more completely representative of Pakistan’s population and includes Azad Jammu and Kashmir (AJK) and the former Federally Administered Tribal Areas (FATA), which were not included in the PDHS 2012–13 for this survey [10]. The 2017–18 PDHS outcomes are indicative at the national level and separately for urban and rural areas. Moreover, the 2017–2018 survey provides more descriptive data on each of the four provinces, namely, Punjab, Sindh, Khyber Pakhtunkhwa, and Balochistan, and also includes AJK, Gilgit-Baltistan (GB), Capital Territory of Islamabad (ICT), and FATA within the sample set. The response rate for this survey was 96%.

The PDHS survey is a joint effort and includes the participation of international agencies. The chief implementation institute for this effort is the National Institute of Population Studies (NIPS), while technical help was provided from ICF International whose funding was provided by the United States Agency for International Development (USAID), and logistical support was provided by the United Nations Population Fund (UNFPA). The Pakistan Bureau of Statistics also assisted in the form of a selection of sample and household listing for the primary sampling units [11]. The survey design followed by the PDHS 2017–18 was a stratified two-stage sampled design. In this sample design, the population is split into groups and new samples are taken from each cluster sample [12], which in this case was achieved by separating each of the eight regions of Pakistan into urban and rural areas. The total sampling strata created were 16 and samples from each stratum were collected using a two-stage selection process.

### Variables

The independent variables used for this study included demographic, social, and economic factors which are closely related to the EBF duration; thus the factors identified as independent variables for this study included **demographic factors**: maternal age, maternal education, maternal occupation, husband’s education, husband’s occupation, family income/ socio-economic status, place of residence (urban/rural), gender of baby, media exposure, pre-lacteal feeds; **health sector variables**: hospital practices, nursing practices, place of delivery, method of delivery, antenatal visits, mothers perceived size of newborn and **socio-cultural factors**: cultural beliefs, family system/ joint family system. A total of 19 independent variables were selected for the study, and the dependent variable is the exclusive breastfeeding duration, which by nature is a continuous variable.

### Statistical methods

We would first use the binary logistic regression model for our parametric estimate and use it to further test for the estimation of the parameters; we used the following specification for this regression [13]:

$$ \uppi =\frac{\exp \left({\upbeta}_0+{\upbeta}_1{\mathrm{X}}_1+\dots +{\upbeta}_{\mathrm{k}}{\mathrm{X}}_{\mathrm{k}}\right)}{1+\exp \left({\upbeta}_0+{\upbeta}_1{\mathrm{X}}_1+\dots +{\upbeta}_{\mathrm{k}}{\mathrm{X}}_{\mathrm{k}}\right)} $$, 0 < π < 1 [1].

For the estimation of parameters in this logistic regression, we use the maximum likelihood estimation (MLE) method. To contrast our findings, we use survival analysis to predict the duration of EBF against 19 independent variables. We use the log-likelihood, AIC, BIC criteria to determine if parametric or non-parametric distribution for the survival analysis would provide better results. Based on these results, we chose the Inverse Gaussian (Weibull) distributions as the most suitable fit for modeling EBF against time.

The data were analyzed using Stata version 15, and a descriptive analysis was completed before running the univariate and regression analysis on the dataset. For the univariate analysis, OR and 95% CI were calculated to assess the impact of the selected factors on EBF, while regression analysis included OR, 95% CI, and *p*-values. The subsequent results were considered significant when *p* < 0.05.

## Results

### Demographic information

The descriptive analysis of the data set is first presented and shows the pattern of data related to the duration of exclusive breastfeeding. For the descriptive analysis, we use the median, frequency and percentage for each selected factor in the study, the results are presented in Table 1.

**Table 1:** Descriptive Analysis

Sr. No.	Factors	Categories	Codes	n(%)	Median
**1**	Maternal Age	15–19	1	96 (7)	2
		20–34	2	1139 (81)	
		35–39	3	177 (12)	
**2**	Region	Punjab	1	579 (41)	2
		Sindh	2	361 (26)	
		KPK	3	314 (22)	
		Balochistan	4	158 (11)	
**3**	Maternal Education	Uneducated	0	593 (42)	1
		Educated	1	819 (58)	
**4**	Husband’s Education	Uneducated	0	332 (24)	1
		Educated	1	1080 (76)	
**5**	Wealth Index	Poor	1	438 (31)	2
		Middle	2	307 (22)	
		Richest	3	667 (47)	
**6**	Respondent Working	No	0	1277 (90)	0
		Yes	1	135 (10)	
**7**	Husband’s Occupation	Unemployed	0	47 (3)	1
		Employed	1	1365 (97)	
**8**	Place of Residence	Urban	1	721 (51)	2
		Rural	2	691 (49)	
**9**	Prenatal Visit to Doctor	No	0	80 (6)	1
		Yes	1	1332 (94)	
**10**	Antenatal Care by Private Doctors	No	0	543 (38)	1
		Yes	1	869 (62)	
**11**	Assistance at Delivery by Doctor	No	0	420 (30)	1
		Yes	1	992 (70)	
**12**	Place of Delivery	Home	1	326 (23)	2
		Govt. Sector	2	379 (27)	
		Private Sector	3	707 (50)	
**13**	Size of Child at Birth	Large	1	87 (6)	2
		Average	2	1017 (72)	
		Small	3	308 (22)	
**14**	Gender of Child	Male	1	674 (48)	2
		Female	2	738 (52)	
**15**	Watching Television	No	0	543 (38)	1
		Yes	1	869 (62)	
**16**	Delivery by Caesarean Section	No	0	1033 (73)	0
		Yes	1	379 (27)	
**17**	Preceding Birth Interval (Months)	Less than 24 months	1	307 (30)	2
		More than or Equal to 24 Months	2	729 (70)	
**18**	Number of Living Children	Less than or Equal to 5 Children	1	1298 (92)	1
		More than 5 Children	2	114 (8)	
**19**	During Antenatal Care Advised on Exclusive Breastfeeding	No	0	652 (46)	1
		Yes	1	760 (54)	

81% of mothers aged between 20 and 34 understudy practiced EBF for six months or less. The median value for the variable residence shows that the respondent belonging to the rural areas and the majority of 41% belongs to Punjab. The average number of respondents visit doctors for prenatal care (*n* = 1332(94%)) and take antenatal care from a private doctors (*n* = 869(62%)). The descriptive results show that the average number of the respondents prefer the government sector hospitals/health center for delivery but the most numbers of respondents *n* = 707 about 50% prefer private hospital for delivery and the size of the child perceived by most of the mothers at the time of delivery was normal (average)(*n* = 1017(72%)). EBF practicing mothers are from the middle class on average and, a major portion of husbands are educated (76%) and employed (97%). On average mothers who exclusively breastfeeding their children are educated but not working and watch television (62%). Advice on exclusive breastfeeding during antenatal care visits (54%) and respondents who do not undergo cesarean section (73%); seemed to be favorable for EBF practice.

### Univariate analysis

After presenting the descriptive analysis, we use univariate and multivariate logistic regression to measure the relationship between exclusive breastfeeding and its factors. Before presenting the results of the binary regression, we use univariate analysis to demonstrate the frequency of EBF with the selected factors.

The results of the univariate analysis show that the odds ratios for the region, maternal education, wealth index, size of a child, watching television, and delivery by cesarean are significant at the 5% level of significance while the rest of the factors are not significant with EBF.

### Binary logistic regression

The binary logistic regression is applied when the dependent variables are dichotomous [13]; in this case, the dependent variable is binary in terms of yes and no response for whether mother practiced EBF whole six months and sample size found is 742. The resulting odd ratios are calculated using the maximum likelihood estimation (MLE) method.

The results of the binary regression model display the odds ratios, *p*-values, and corresponding 95% confidence intervals for each different factor related to EBF. The calculated values of odd ratios for maternal age, region, maternal education, watching television, and child-size have significant *p*-values while all the rest of the factors have insignificant odd ratios. It can also be observed from the Pseudo R-Square and Chi-square values that the model is overall significant.

### Survival analysis

Survival analysis is used to measure data before one or more events of interest occur, where the predictor variable is the one. Survival analysis is a collection of techniques used to analyze the data that persists before an occurrence happens and its relevant factors [14]. It is a collection of statistical procedures for data analysis for which the outcome variable of interest is time until an event occurs; in this case, it is the duration of exclusive breastfeeding. In comparison to only binary response, survival analysis can also gauge the duration lesser than six months up till which respondent practiced EBF and sample size found to be 1412.

We use AIC and BIC to determine the model selection criteria for the use of either parametric or non-parametric distributions for the survival analysis. The AIC is a widely accepted measure of goodness of fit for nay statistical model while the BIC is a type of model selection among a class of parametric models with a different number of parameters [15]. Table 2 shows the results of the AIC and BIC applied for this check; the selection of either a parametric or non-parametric survival distribution is based on these results:

**Table 2:** Information Criteria for selection of models

Information Criteria	PDHS 2017–18
Parametric Survival Models	
AIC	768.648
BIC	855.312
Non-Parametric Models	
AIC	2623.229
BIC	2701.64

The calculated values of AIC and BIC for parametric and semi-parametric survival models for the duration of exclusive breastfeeding are minimum for the parametric model; it is thus confirmed that the parametric survival model would provide a better fit for EBF duration.

The potential parametric survival models which can be used to model the duration of exclusive breastfeeding are Exponential, Weibull, Gamma Frailty, Gompertz, Loglogistic, Lognormal, and Inverse Gaussian are shown in Table 3. The measures which are used to specify the best parametric survival model for the duration of exclusive breastfeeding are log-likelihood, AIC and BIC.

**Table 3:** Goodness of Fit Test to Test the Appropriate Model for Duration of Exclusive Breastfeeding PDHS 2017–18

Hypothesized Model	LL	Chi-square	p-value	AIC	BIC
Exponential	−434.93	616.12	0.00***	907.86	986.27
Weibull	−372.67	371.64	0.00***	785.35	867.88
Gompertz	−385.49	378.97	0.00***	810.98	893.52
Loglogistic	−372.31	962.29	0.00***	784.62	867.16
lognormal	−366.93	898.91	0.00***	773.87	856.40
Gamma Frailty (Weibull)	−371.82	301.19	0.00***	785.65	872.31
Inverse Gaussian (Weibull)	−363.32	119.70	0.00***	768.64	855.31

*** *p* < 0.01, ** *p* < 0.05, * *p* < 0.1

Based on the results presented above, we determine that the best parametric model fit for the duration of EBF and its associated factors is the Inverse Gaussian (Weibull) distribution. The results are presented in Table 4.

**Table 4:** Parametric Estimation of Exclusive Breastfeeding Duration using Inverse Gaussian (Weibull)

Factors	Hazard Ratio	p-value
**Maternal Age**		
15–19	0.0635	0.006***
20–34	0.2802	0.000***
35–39	0.4080	0.000***
**Region**		
Punjab	0.0479	0.000***
Sindh	0.2937	0.000***
KPK	0.4463	0.000***
Balochistan	0.5717	0.000***
**Maternal Education**		
Uneducated	1.0000	–
Educated	0.0504	0.000***
**Husband’s Education**		
Uneducated	1.0000	–
Educated	0.0734	0.000***
**Wealth Index**		
Poor	0.0979	0.000***
Middle	0.2705	0.000***
Richest	0.3884	0.000***
**Respondent Working**		
No	1.0000	–
Yes	0.0393	0.000***
**Husband’s Occupation**		
Unemployed	1.0000	–
Employed	0.0749	0.000***
**Place of Residence**		
Urban	0.0755	0.000***
Rural	0.2758	0.000***
**Prenatal Visit to Doctor**		
No	1.0000	–
Yes	0.0778	0.000***
**Antenatal Care by Private Doctors**		
No	1.0000	–
Yes	0.0754	0.000***
**Assistance at Delivery by Doctor**		
No	1.0000	–
Yes	0.0730	0.000***
**Place of Delivery**		
Home	0.0887	0.000***
Govt. Sector	0.2880	0.000***
Private Sector	0.4012	0.000***
Large	0.0117	0.000***
Average	0.2814	0.000***
Small	0.4315	0.000***
**Gender of Child**		
Male	0.0777	0.000***
Female	0.2722	0.000***
**Watching Television**		
No	1.0000	–
Yes	0.0603	0.000***
**Delivery by Caesarean Section**		
No	1.0000	–
Yes	0.0495	0.000***
**Preceding Birth Interval (Months)**		
Less than 24 months	0.0764	0.000***
More than or Equal to 24 Months	0.2770	0.000***
**Number of Living Children**		
Less than or Equal to 5 Children	0.0720	0.000***
More than 5 Children	0.3390	0.001***
**During Antenatal Care Advised on Exclusive Breastfeeding**		
No	1.0000	−`
Yes	0.0583	0.000***

*** *p* < 0.01, ** *p* < 0.05, * *p* < 0.1

The results show that all the socio-economic and demographic factors are significantly associated with duration of exclusive breastfeeding at a 5% level of significance except maternal education (uneducated), husband education (uneducated), respondent working (no), husband’s occupation (unemployed), a prenatal visit to the doctor (no), antenatal care by the private doctor (no), assistance at delivery by the doctor (no), watching television (no), delivery by cesarean (no), and during antenatal Care Advised on Exclusive Breastfeeding (no).

## Discussion

The results presented in this paper show which demographic, social, and economic factors are significantly related to exclusive breastfeeding practices in Pakistan. The significant factors identified within this study include maternal age, region, maternal education, watching television, and child size. Additionally, our paper presents the results of the survival analysis where we use parametric techniques to model the duration of EBF against its factors. The comparison shows that the results of the parametric survival analysis are better predictors of EBF and its factors than the results of the binary logistic regression.

These results can be used to identify the reasons why EBF levels remain low in Pakistan and subsequently, compromise infant and maternal health. Identification of these factors allows health and education policy ministries to actively target these factors so that child health indices within the country can be improved; to this end, the appropriate departments and institutes need to devise national and provincial level strategies to combat the ill-effects of improper child nutrition and feeding practices.

### Significant factors and impact on EBF

It is widely acknowledged that education plays the most important role in enhancing or compromising infant health. The low levels of literacy in Pakistan combined with high rates of poverty, especially in rural areas, are a major hindrance towards proper child nutrition practices [16]. Low literacy levels often prove dangerous to the feeding practices for the infant; for instance, in rural areas, it is a common cultural and societal belief that breastfeeding should be delayed because many consider that colostrum is stale milk and fresh milk would arrive on the third day after birth [17]. During this waiting period, the newborn is usually fed traditional and ceremonial foods such as honey, glucose, and butter mixed with sugar, which is a dangerous practice since such liquids contain a high risk of contamination [1]. Problems such as these can be alleviated by proper information regarding infant care and nutrition in rural areas, especially in the regions of Balochistan, Khyber Pakhtunkhwa, and Sindh. However, the problems of illiteracy and poverty are rooted in more systemic problems [18]; regardless, the condition can be improved by a targeted health campaign directed solely towards raising awareness about proper childcare feeding practices. This can include specific messages provided to women about breastfeeding as an important source of nutrition for their child and can highlight some of the dangerous impacts of traditional and cultural feeding practices which are considered safe and healthy [19]. Mothers should also be encouraged to breastfeed as long as possible and should be educated about the protective benefits of colostrum and the hazardous habits of feeding infants supplements other than breast milk should be strongly discouraged.

There have been similar studies conducted within Pakistan that have identified a host of factors that contributes towards unhealthy feeding practices among infants and children. Another study identified several important demographic and socioeconomic factors; these factors were breastfeeding status, age, number of living children, residence, education, wealth, information sources about breastfeeding practices, assistance during delivery, and place of delivery. The study used a multiple logistic regression analysis to highlight the significant factors for breastfeeding practices in Pakistan, whereas this study uses comprehensive and comparative statistical measures to study a range of demographic and socio-economic factors and their relationship with breastfeeding practices [20].

Our study also supports a variety of previous studies which found maternal education to be an important factor for determining infant health and breastfeeding practices. This is despite the perception that, in Pakistan, educated and wealthy women located in urban areas are likely to stop breastfeeding earlier, as compared to women located in rural areas [21]. This practice of supplemental feedings and early stoppage of exclusive breastfeeding could have a significant trickling down effect since women who belong to the higher income classes are often role models for women who belong to the lower-income class groups. Regardless, the results do show that educated women are likely to breastfeed for a longer duration than uneducated women. Our results also show that women who watch television are likely to breastfeed for a longer duration; these findings are correlated with those of education since information regarding breastfeeding and proper nutrition can be gained from television. However, watching television can also prove to be detrimental to the duration of EBF since.

Marketing campaigns for formula milk are often presented very attractively; this is contradictory to the practice of EBF and results in a gap between knowledge and practice regarding breastfeeding practices in the country [22].

Our study finds that the size of the child is also a significant factor in EBF duration and that women with smaller babies at birth tend to breastfeed for a longer duration. This is because children who are perceived large at birth are less likely to develop undernutrition problems later in life as compared to children who are perceived as small at the time of birth [23]; thus, mother’s perception of the size of the child is a cause of bias towards the signs of nutrition and may lead to unhealthy feeding habits as a result of this perception, including a reduced duration of EBF.

Proper nutrition and feeding habits for infants should also be dispensed at hospitals and health service providers so that staff can educate mothers about infant nutrition needs and the role of breastfeeding. Additionally, the duration of EBF can also be increased in Pakistan through community health workers, who can provide counseling to women during antenatal, natal, and postnatal home visits, since the majority of births in Pakistan take place at home [24]. Health care policy should also seek to dismantle traditions and cultural norms which are harmful towards EBF practices; this can be achieved by public service messages addressed towards women located in both the urban and rural areas of Pakistan. Mothers should be made aware that there is no need for supplemental breastfeeding during the first five months and should be informed that the infant requires only breast milk for all of its nutrition needs during this time [25].

Some of the adverse effects of changing lifestyles, supplementary feeding practices, and perceived insufficiency of breastfeeding should also be reduced or targeted through public service health campaigns and messages. These campaigns need to focus on women across all groups of society, with special emphasis on rural and lower-middle-income class groups.

There is also a dire need to improve the health care infrastructure of Pakistan, which is a primary factor towards the contribution of infant malnutrition and suboptimal infant feeding practices. The government needs to address this on an urgent basis since the situation of health care facilities in rural areas is poor and in need of critical attention. Maternal care centers in rural areas are sadly deprived and suffer from an acute shortage of medical staff, equipment, information, and infrastructure [26]. These conditions need to be drastically improved if Pakistan’s statistics regarding maternal and infant care are to be amended.

Some limitations of this study include are that it focused on secondary data and is thus restricted by previously settled sample size structure and questionnaires. Several factors cause the duration of exclusive breastfeeding, but only selected factors were the subject of this research. Also, this study uses a cross-sectional design, which limits its ability to establish causation among the variables. A study that uses a time-series analysis would be able to identify cause-and-effect relationships among the important independent variables. Lastly, this paper uses a quantitative study design, which does not adequately explore the traditional norms and values pervading the society which are often the most acute determinants of child care and nutrition. Future studies can thus apply qualitative and mixed designs to incorporate these effects into the investigation of breastfeeding practices in Pakistan. This can also be accomplished by applying a broader dataset towards the question at hand and can use quantile regression to enhance results.

## Conclusion

The evidence for EBF as a source of proper nutrition for infants and children, and the risks for discontinuing this practice is well-established and documented. A duration of breastfeeding which is less than recommended leads to malnutrition, stunting, and infant mortality. Likewise, the practices of bottle-feeding or supplemental feeding in the first six months of birth are proved to be detrimental to child development and nutrition. In Pakistan, the practice of EBF is suboptimal and is heavily subject to the prevailing social, economic, and demographic factors, which frequently determine the duration of exclusive breastfeeding practice. This paper, therefore, identifies which of these social, economic, and demographic factors have the most impact on the exclusive breastfeeding duration in Pakistan from data collected by the Pakistan Demographic and Health Survey 2017–18. The results of the subsequent binary logistic regression find that region, maternal education, maternal age, watching television, and size of a child have the most significant impact on the practice of EBF. However, the factors are better predicted when we use parametric survival analysis to determine our results, consequently, the use of the survival analysis finds that all of the factors shave a significant association with EBF. Contrarily, Many important proved factors from different researches such as residence, wealth index, husband education, respondent working, husband’s occupation, a prenatal visit to the doctor, antenatal care by a private doctor, assistance at delivery by the doctor, place of delivery, delivery by cesarean, child gender, Preceding Birth Interval, number of living children and during antenatal Care Advised on Exclusive Breastfeeding are found to be insignificant using binary logistic regression. These results should help policy and health officials to determine which factors to target for improving the overall health system infrastructure in Pakistan, and should also direct towards developing public service campaigns that target women across the country about proper feeding practices for infants and children.

## Supplementary Information

**Additional file 1.**

## Data Availability

The data is available publically at “The DHS Program” website: https://dhsprogram.com/data/dataset/Pakistan_Standard-DHS_2017.cfm?flag=0

## References

[1] Syeda B, Agho K, Wilson L, Maheshwari GK, Raza MQ (2021). Relationship between breastfeeding duration and undernutrition conditions among children aged 0–3 years in Pakistan. Int J Pediatr Adolesc Med.

[2] Joseph FI, Earland J (2019). A qualitative exploration of the sociocultural determinants of exclusive breastfeeding practices among rural mothers, North West Nigeria. Int Breastfeed J.

[3] Zakar R, Zakar MZ, Zaheer L, Fischer F (2018). Exploring parental perceptions and knowledge regarding breastfeeding practices in Rajanpur, Punjab Province, Pakistan. Int Breastfeed J.

[4] Velusamy V, Premkumar PS, Kang G (2017). Exclusive breastfeeding practices among mothers in urban slum settlements: pooled analysis from three prospective birth cohort studies in South India. Int Breastfeed J.

[5] Kim Y-M, Haq ZU, Soomro J, Sultana Z, Faizunnisa A, Agha S (2015). Case study: effects of a media campaign on breastfeeding behaviors in Sindh Province, Pakistan. World Health Popul.

[6] Oakley L, Baker CP, Addanki S, Gupta V, Walia GK, Aggarwal A, Bhogadi S, Kulkarni B, Wilson RT, Prabhakaran D, Ben-Shlomo Y, Davey Smith G, Radha Krishna KV, Kinra S (2017). Is increasing urbanicity associated with changes in breastfeeding duration in rural India? An analysis of cross-sectional household data from the Andhra Pradesh children and parents study. BMJ Open.

[7] Pretorius, C. E. et al. Exclusive Breastfeeding, Child Mortality, and Economic Cost in Sub-Saharan Africa. Pediatrics 147, doi:10.1542/peds.2020-030643 (2021). 10.1542/peds.2020-030643 33622796

[8] Ogbo FA, Page A, Idoko J, Agho KE (2018). Population attributable risk of key modifiable risk factors associated with non-exclusive breastfeeding in Nigeria. BMC Public Health.

[9] Bank, T. W Demographic and health survey 2017–2018 Pakistan,. (2018).

[10] Program, T. D Pakistan Demographic and Health Survey, <https://dhsprogram.com/Countries/Country-Main.cfm?ctry_id=31&c=Pakistan&Country=Pakistan&cn=&r=4> (2018).

[11] Studies, N. I o. P. Pakistan Demographic and Health Survey (PDHS) 2017–18. 2–20 (Islamabad, Pakistan, 2017).

[12] Fuller, W. A. Sampling statistics. Vol. 560 (John Wiley & Sons, 2011).

[13] Hilbe, J. M. Logistic regression models. (CRC press, 2009).

[14] Kleinbaum, D. G. & Klein, M. Survival analysis. (Springer, 2010).

[15] Burnham KP, Anderson DR (2004). Multimodel inference: understanding AIC and BIC in model selection. Sociol Methods Res.

[16] Hanif HM (2011). Trends in breastfeeding and complementary feeding practices in Pakistan, 1990-2007. Int Breastfeed J.

[17] Morisky DE (2002). Breastfeeding practices in Pakistan. Pak J Nutr.

[18] Hazir T, Akram DS, Nisar YB, Kazmi N, Agho KE, Abbasi S, Khan AM, Dibley MJ (2013). Determinants of suboptimal breast-feeding practices in Pakistan. Public Health Nutr.

[19] Saeed OB, Haile ZT, Chertok IA (2020). Association between exclusive breastfeeding and infant health outcomes in Pakistan. J Pediatr Nurs.

[20] Noh J-W, Kim YM, Akram N, Yoo KB, Cheon J, Lee LJ, Kwon YD, Stekelenburg J (2019). Factors affecting breastfeeding practices in Sindh Province, Pakistan: a secondary analysis of cross-sectional survey data. Int J Environ Res Public Health.

[21] Sabin A, Manzur F, Adil S (2017). Exclusive breastfeeding practices in working women of Pakistan: a cross sectional study. Pakistan journal of medical sciences.

[22] Hanif R, Khalil E, Sheikh A, Harji A, Haris S, Rasheed MW, Ahmed S, Abdul Aziz K, Shaheen E, Younus A, Mansoor M, Hameed F, Touseef M, Yaseen T (2010). Knowledge about breastfeeding in accordance with the national policy among doctors, paramedics and mothers in baby-friendly hospitals. JPMA The Journal of the Pakistan Medical Association.

[23] Hasnain S, Hashmi S (2009). Consanguinity among the risk factors for underweight in children under five: a study from rural Sindh. J Ayub Med Coll.

[24] Bashour HN, Kharouf MH, AbdulSalam AA, el Asmar K, Tabbaa MA, Cheikha SA (2008). Effect of postnatal home visits on maternal/infant outcomes in Syria: a randomized controlled trial. Public Health Nurs.

[25] Salasibew M, Kiani A, Faragher B, Garner P (2008). Awareness and reported violations of the WHO international code and Pakistan's national breastfeeding legislation; a descriptive cross-sectional survey. Int Breastfeed J.

[26] Das JK (2020). Impact of conflict on maternal and child health service delivery–how and how not: a country case study of conflict affected areas of Pakistan. Confl Heal.

