# Supplementary material for: Factors influencing exclusive breastfeeding duration in Pakistan: a population-based cross-sectional study
Source: BMC Public Health. 2021 Nov 3;21:1998. doi: 10.1186/s12889-021-12075-y (PMC8567599; doi:10.1186/s12889-021-12075-y)
Supplement: Supplementary file 1 — Additional file 1. [file 12889_2021_12075_MOESM1_ESM.docx]

**Supplementary Tables**

**S 1:** Univariate analysis of Socio-Economic and Demographic Factors of Exclusive Breastfeeding using Binary Logistic Regression

| **Independent Variables** | **Exclusive Breastfeeding** | | **Univariate Analysis**  **OR (95% CI)** |
| --- | --- | --- | --- |
|  | No (343) | Yes (399) |  |
| **Maternal Age** | | | |
| 15-19 | 27 | 30 | 0.921  (0 .671, 1.265) |
| 20-34 | 266 | 319 |  |
| 35-39 | 50 | 50 |  |
| **Region** | | | |
| Punjab | 163 | 98 | 1.441  (1.245, 1.669)*** |
| Sindh | 83 | 139 |  |
| KPK | 59 | 109 |  |
| Balochistan | 38 | 53 |  |
| **Maternal Education** |  |  |  |
| Uneducated | 124 | 217 | 0 .474  (0.353,0 .638)*** |
| Educated | 219 | 182 |  |
| **Husband’s Education** |  |  |  |
| Uneducated | 76 | 106 | 0 .786  (0.561, 1.103) |
| Educated | 267 | 293 |  |
| **Wealth Index** |  |  |  |
| Poor | 110 | 154 | 0.848  (0.720,0.999)** |
| Middle | 74 | 85 |  |
| Richest | 159 | 160 |  |
| **Respondent Working** |  |  |  |
| No | 320 | 361 | 1.464  (0.854, 2.511) |
| Yes | 23 | 38 |  |
| **Husband’s Occupation** |  |  |  |
| Unemployed | 9 | 18 | 0.570  (0.252, 1.286) |
| Employed | 334 | 381 |  |
| **Place of Residence** |  |  |  |
| Urban | 165 | 196 | 0.960  (0.719, 1.281) |
| Rural | 178 | 203 |  |
| **Prenatal Visit to Doctor** |  |  |  |
| No | 21 | 22 | 1.117  (0.603, 2.069) |
| Yes | 322 | 377 |  |
| **Antenatal Care by Private Doctors** |  |  |  |
| No | 129 | 170 | 0.812  (0.604, 1.090) |
| **S 1:** Univariate analysis of Socio-Economic and Demographic Factors of Exclusive Breastfeeding using Binary Logistic Regression (Continue) | | | |
| **Independent Variables** | **Exclusive Breastfeeding** | **Univariate Analysis** | **Independent Variables** |
| Yes | 214 | 229 |  |
| **Assistance at Delivery by Doctor** |  |  |  |
| No | 100 | 125 | 0.902  (0.658, 1.235) |
| Yes | 243 | 274 |  |
| **Place of Delivery** |  |  |  |
| Home | 78 | 104 | 0.931  (0.781, 1.110) |
| Govt. Sector | 0 | 102 |  |
| Private Sector | 171 | 193 |  |
| **Size of Child at Birth** |  |  |  |
| Large | 19 | 22 | 0.645  (0.478,0.869)*** |
| Average | 232 | 312 |  |
| Small | 92 | 65 |  |
| **Gender of Child** |  |  |  |
| Male | 164 | 181 | 1.103  (0.826, 1.473) |
| Female | 179 | 218 |  |
| **Watching Television** |  |  |  |
| No | 110 | 194 | 0.498  (0.369,0.673)*** |
| Yes | 233 | 205 |  |
| **Delivery by Caesarean Section** |  |  |  |
| No | 242 | 316 | 0.629  (0.450,0.879)*** |
| Yes | 101 | 83 |  |
| **Preceding Birth Interval (Months)** |  |  |  |
| Less than 24 months | 77 | 75 | 1.331  (0.917, 1.931) |
| More than or Equal to 24 Months | 182 | 236 |  |
| **Number of Living Children** |  |  |  |
| Less than or Equal to 5 Children | 314 | 356 | 1.307  (0.797, 2.145) |
| More than 5 Children | 29 | 43 |  |
| **During Antenatal Care Advised on Exclusive Breastfeeding** |  |  |  |
| No | 159 | 199 | 0.868  (0.650, 1.159) |
| Yes | 184 | 200 |  |
| *** p<0.01, ** p<0.05, * p<0.1 |  |  |  |

**S 2:** Binary Logistic Regression Analysis

| **Independent Variable** | **Odds Ratios** | **p-value** | **95 % C.I** |
| --- | --- | --- | --- |
| Maternal Age | 0.619 | 0.053* | (0.381, 1.006) |
| Region | 1.349 | 0.003*** | (1.11, 1.639) |
| Residence | 1.053 | 0.81 | (0.689, 1.611) |
| Maternal Education | 0.501 | 0.002*** | (0.324, 0.776) |
| Watching Television | 0.706 | 0.086* | (0.474, 1.051) |
| Wealth Index | 1.074 | 0.624 | (0.808, 1.428) |
| Husband’s Education | 1.001 | 0.996 | (0.631, 1.589) |
| Husband’s Occupation | 0.664 | 0.442 | (0.233, 1.888) |
| Respondent Working | 1.042 | 0.9 | (0.549, 1.977) |
| Prenatal Visit to Doctor | 1.462 | 0.35 | (0.659, 3.242) |
| Assistance at Delivery | 0.971 | 0.924 | (0.527, 1.788) |
| Delivery Place | 1.161 | 0.395 | (0.823, 1.637) |
| Delivery By Caesarean | 0.761 | 0.241 | (0.482, 1.201) |
| Child Size | 0.627 | 0.011** | (0.437, 0.899) |
| Antenatal Care | 0.76 | 0.156 | (0.52, 1.11) |
| Child Gender | 1.075 | 0.688 | (0.755, 1.53) |
| Preceding Birth Interval | 1.166 | 0.455 | (0.779, 1.748) |
| Antenatal Care Advised | 1.112 | 0.572 | (0.77, 1.604) |
| Number of Living Children | 1.147 | 0.657 | (0.626, 2.104) |
| Constant | 3.734 | 0.282 | (0.339, 41.12) |
| Pseudo R-squared | 0.075 |  |  |
| Chi-square | 58.801 |  |  |
| Prob > chi2 | 0.000 |  |  |
| AIC | 766.637 |  |  |
| BIC | 853.550 |  |  |
| *** p<0.01, ** p<0.05, * p<0.1 | | | |
